# Complete mitochondrial genome of the giant root-rat (*Tachyoryctes macrocephalus*)

**DOI:** 10.1080/23802359.2021.1944388

**Published:** 2021-07-01

**Authors:** Victoria M. Reuber, Alba Rey-Iglesia, Michael V. Westbury, Andrea A. Cabrera, Nina Farwig, Mikkel Skovrind, Radim Šumbera, Tilaye Wube, Lars Opgenoorth, Dana G. Schabo, Eline D. Lorenzen

**Affiliations:** a Department of Biology, Conservation Ecology, University of Marburg, Marburg, Germany; b Section for Evolutionary Genomics, GLOBE Institute, University of Copenhagen, Copenhagen, Denmark; c Department of Zoology, University of South Bohemia, České Budějovice, Czech Republic; d Department of Zoological Sciences, College of Natural and Computational Sciences, Addis Ababa University, Addis Ababa, Ethiopia; e Department of Biology, Plant Ecology & Geobotany, University of Marburg, Marburg, Germany

**Keywords:** Mitochondrial genome, *Tachyoryctes*, phylogenetics, Bale Mountains

## Abstract

The endangered giant root-rat (*Tachyoryctes macrocephalus*, also known as giant mole rat) is a fossorial rodent endemic to the afro-alpine grasslands of the Bale Mountains in Ethiopia. The species is an important ecosystem engineer with the majority of the global population found within 1000 km^2^. Here, we present the first complete mitochondrial genome of the giant root-rat and the genus *Tachyoryctes*, recovered using shotgun sequencing and iterative mapping. A phylogenetic analysis including 15 other representatives of the family Spalacidae placed *Tachyoryctes* as sister genus to *Rhizomys* with high support. This position is in accordance with a recent study revealing the topology of the Spalacidae family. The full mitochondrial genome of the giant root-rat presents an important resource for further population genetic studies.

The giant root-rat (*Tachyoryctes macrocephalus*, Rüppell, 1842), also known as giant mole rat and big-headed African mole rat, is a fossorial rodent and ecosystem engineer endemic to the Bale Mountains of south-east Ethiopia. The species is naturally confined to afro-alpine grasslands, where it constructs large underground burrow systems (Yalden 1985). Giant root-rats substantially impact their surroundings through a combined effect of soil perturbation and above-ground herbivory, with consequences for vegetation patterns, soil formation, and nutrient availability (Šklíba et al. 2017). In addition to their ecological importance, they are the most important prey of the endangered Ethiopian wolf (*Canis simensis;* Sillero-Zubiri and Gottelli 1995) in the Bale Mountains. Giant root-rats have a limited distribution range across the Bale Mountain massif at elevations ranging between 3000 and 4150 m above sea level, with the majority of the population occurring within 1000 km^2^ (Sillero-Zubiri et al. 1995, Yalden and Largen 1992). The species is prone to human-induced habitat degradation and is currently listed as endangered by the IUCN (Lavrenchenko and Kennerley 2016).

A recent study revealed that giant root-rats were the key food resource of prehistoric hunter gatherers inhabiting rock shelters in the Bale Mountains 47,000–31,000 years ago. The consumption of the species facilitated the occupation of the world’s earliest known high-altitude residential site (Ossendorf et al. 2019), and suggests that the giant root-rat population has been shaped by human activities across millennia.

To date, genetic studies on the giant root-rat are limited. However, one study presented a phylogeny of *Tachyoryctes* based on two mitochondrial genes (cytochrome b, and cytochrome c oxidase subunit I) and three nuclear genes (NAD synthetase 1, wntless, and recombination activating gene 1) (Šumbera et al. 2018). The analysis revealed a split between the giant root-rat and one lineage of the African root-rat (*T. splendens 1* sensu, Šumbera et al. 2018), about 1.3 million years ago. Another recent study revealed the topology of the entire Spalacidae family (He et al. 2020). Complete mitochondrial genomes are still lacking for the *Tachyoryctes* genus.

We collected a tissue sample from a live giant root-rat individual in February 2020, from the eastern part of the Sanetti Plateau of the Bale Mountains National Park (6°52′24.5N, 39°52′25.4E), under permits from the Ethiopian Wildlife Conservation Association. The voucher tissue sample of *T. macrocephalus* was deposited at the Conservation Ecology group, University of Marburg (www.uni-marburg.de, Dana G. Schabo, dana.schabo@staff.uni-marburg.de) and DNA extracts at the GLOBE Institute, University of Copenhagen (Denmark; voucher number: CGG_1_024459). The DNA was extracted using the Qiagen DNeasy® Blood and Tissue Kit, and sheared to approximately 400 bp using the Covaris M220 Focused-ultrasonicator. DNA fragments were built into an Illumina library following the protocol from Carøe et al. (2018), and sequenced on an Illumina HiSeq 4000. We trimmed the adapter sequences from the raw reads using Skewer v0.2.2 (Jiang et al. 2014), and removed PCR duplicates of 100% identity using Prinseq-lite v0.20.4 (Schmieder and Edwards 2011). For the assembly of the complete mitochondrial genome, we followed an iterative approach using MITObim v1.8 (Hahn et al. 2013) with default parameters, 51 k-bait and a mismatch value of 3. We repeated this analysis five times independently using one of the five available Spalacidae mitochondrial genomes as reference sequence (GenBank accession KC789518.1, NC_020756.1, JN540033.1, JX014234.1 and JN571130.1). We imported the resultant alignment files into Geneious Prime 2021 and created five consensus sequences (one for each reference) with a minimum read depth of 20× and a 75% consensus base call threshold. All five consensus sequences were aligned using MAFFT v7.392 (Katoh and Standley 2013) and were manually inspected for mismatches and to find point of circularity. All consensus sequences were near identical, regardless of mapping reference with the exception of some insertions/deletions (indels). As indels were not shared between multiple reference sequences, they were excluded from the final consensus sequence.

We obtained a 16,646 bp sequence length (GenBank accession MW751806) and conducted an annotation using MITOS (Bernt et al. 2013), which uncovered all protein-coding genes, tRNAs, and rRNAs typical for vertebrate mitochondrial genomes. We compiled a maximum-likelihood phylogenetic tree with 15 mitochondrial genomes available for the Spalacidae family, and specified *Nannospalax galili* as outgroup. The analysis was conducted using RaxML-HPC2 on XSEDE v8.2.12 (Stamatakis 2014) on the Cipres server (Miller et al. 2010), and run with default parameters.

Our phylogenetic tree places the giant root-rat as sister clade to the genus *Rhizomys* – which are also fossorial rodents (Wilson et al. 2017) – with high support, in accordance with a phylogenetic analysis based on mitochondrial and nuclear genes (Figure 1) (Šumbera et al. 2018). Furthermore, our retrieved topology of the Spalacidae family is consistent with the phylogeny recently presented in He et al. (2020). The full mitochondrial genome of the giant root-rat is a vital resource for future population genetic studies of the species.

**Figure 1.**
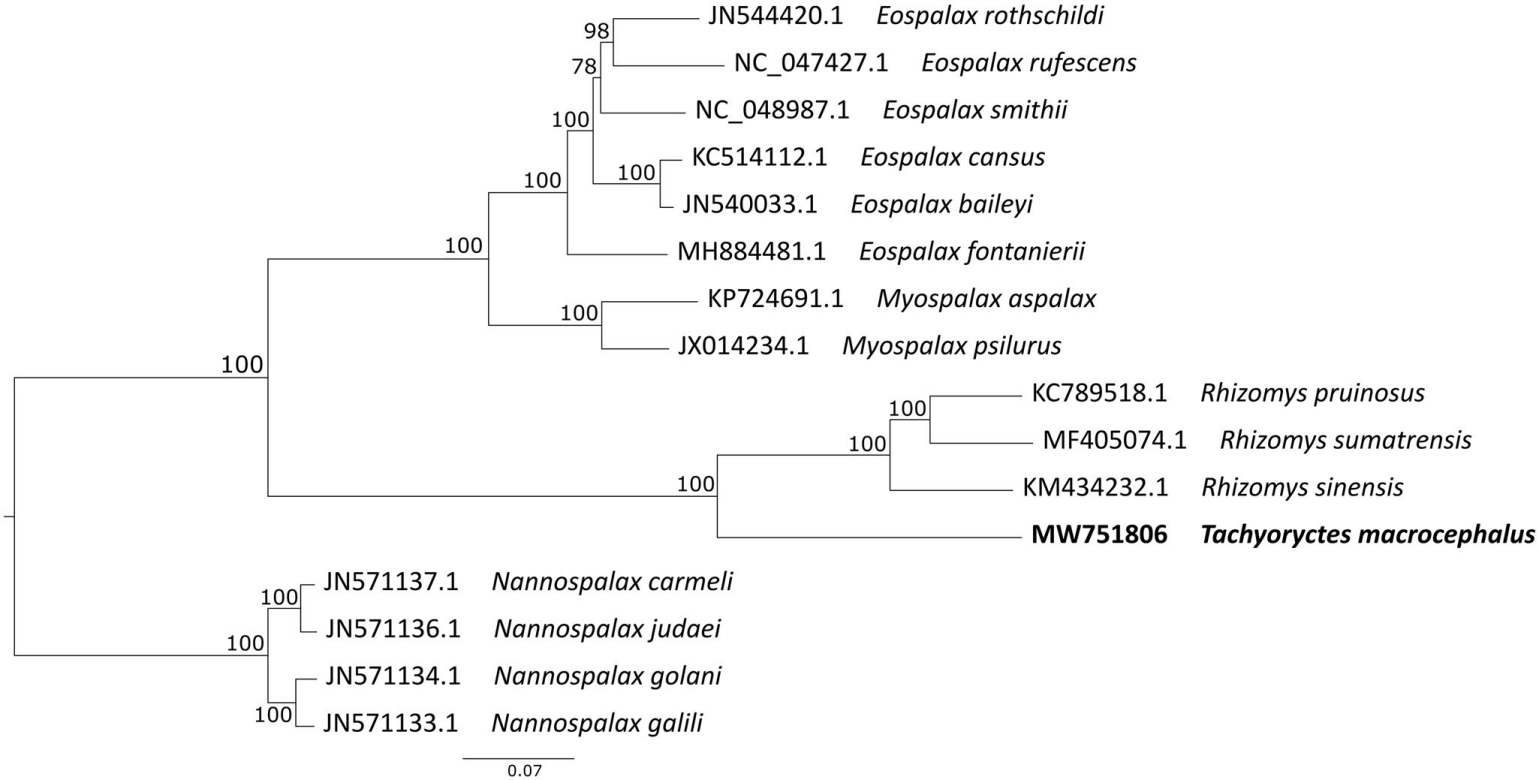
Maximum-likelihood phylogenetic tree showing the relationship between the giant root-rat (*Tachyoryctes macrocephalus*, in bold) and other representatives of the Spalacidae family. The numbers on branches display bootstrap support.

## Data Availability

The data that support the findings of this study are openly available in GenBank of NCBI at https://www.ncbi.nlm.nih.gov under the accession no. MW751806. The associated BioProject, SRA, and Bio-Sample numbers are PRJNA735606, SRX11080558, and SAMN19589957, respectively.
